# Generalized and pregnancy-related anxiety prevalence and predictors among pregnant women attending primary health care in Qatar, 2018–2019

**DOI:** 10.1016/j.heliyon.2020.e05264

**Published:** 2020-10-23

**Authors:** Sarah Naja, Noora Al Kubaisi, Rajvir Singh, Iheb Bougmiza

**Affiliations:** a Hamad Medical Corporation, Doha, Qatar; b Primary Health Care Corporation, Doha, Qatar; c Biostatistics, Hamad Medical Corporation, Doha, Qatar

**Keywords:** Epidemiology, Obstetrics & gynecology, Public health, Clinical psychology, Pregnancy, Social sciences, Anxiety, Pregnancy-specific, EPDS-3A, PRAQ-R2

## Abstract

**Background:**

Cumulative evidence suggests that early identification of anxiety in pregnancy is important, given that antenatal anxiety has been linked to morbid outcomes in expecting mothers and their offspring. However, the burden of antenatal anxiety is not yet known in Qatar. This research aims to measure the prevalence and determinants of generalized and pregnancy-related anxiety among pregnant women.

**Methods:**

Eight hundred pregnant women completed a structured interview and self-administrated questionnaires after being selected through probability sampling from nine primary healthcare centers distributed across Qatar. We subjected the data to Binary and Multiple Logistic Regression Analysis. Furthermore, we conducted a Confirmatory Factor Analysis for the utilized scales.

**Results:**

Out of eight hundred participants, 26.5% reported high pregnancy-related anxiety, while 16.4% had a generalized anxiety disorder. A high level of perceived social support and resilience was shown to mitigate generalized and pregnancy-related anxiety. However, we revealed that different determinants influence the two types of anxiety.

**Limitations:**

There is no recognized optimal cut-off point to distinguish ‘high risk’ in pregnancy-related anxiety scales.

**Conclusions:**

Pregnancy-related anxiety is more prevalent than generalized anxiety among pregnant women in Qatar, indicating that stakeholders must include screening for pregnancy-related anxiety in Qatar's clinical guidelines. Tailored interventional studies could focus on increasing resilience and social support to decrease the burden of antenatal anxiety.

## 1 Introduction

Antenatal anxiety disorders are common (Falah-Hassani et al., 2017), and are significantly higher in pregnancy than the non-pregnant population (39% versus 16%) (Adewuya et al., 2006). Anxiety negatively affects the expecting mothers and their offspring, increases the odds of preterm delivery, and cultivates neurodevelopmental disorders (Rose et al., 2016). These adverse effects led to changes in the guidelines recommending early detection of antenatal anxiety (NICE., 2014; SIGN., 2012). Furthermore, screening and treatment of antenatal anxiety disorder in pregnancy has been shown to be cost-effective (Bauer et al., 2014).

The prevalence of anxiety disorders in pregnancy shows high variability, ranging from 10% in South London (Nath et al., 2018) to 41% in Central America (Verbeek et al., 2015). The reported variation in prevalence is related to heterogeneity in the operational and conceptual definition of anxiety disorders. Recently, a meta-analysis published in 2019 showed that antenatal anxiety disorders affect 20.7% of pregnant women; Confidence Interval *CI* [16.7%–25.4%]. Fawcett et al. (2019) reported generalized anxiety disorder (GAD) to be one of the most prevalent perinatal disorders during pregnancy; other types of antenatal anxiety disorders were described, such as panic attacks, obsessive-compulsive disorder, acute stress disorder, specific phobia, and post-traumatic stress disorder.

Furthermore, Huizink et al. (2004) specified a form of state anxiety specific to pregnancy concerns such as worries about giving birth, having an ill child, and concerns about one's appearance. Moreover, he described this type of antenatal anxiety to be distinctive entitled ‘pregnancy-related anxiety’. Importantly, Dunkel Schetter and Tanner (2012) and Reck et al. (2013) identified pregnancy-related anxiety as the most potent psychological predictor of birth and child development and to be independent of generalized anxiety. Nevertheless, limited studies have investigated pregnancy-related anxiety. Pampaka et al. (2018) reported that 15% of pregnant women had a high level of pregnancy-related anxiety in Kuwait. Whereas Nath et al., 2019 stated that up to 55% of pregnant women, at less than twenty-four weeks of gestation, suffered from a high level of pregnancy-related anxiety in India.

The determinants of antenatal anxiety disorders are many, and they include social, psychological, behavioural, environmental, and biological factors that shape pregnancy and may lead to antenatal anxiety. A systematic review identified that a history of pregnancy loss, a previous affective disorder, and medical complications were significant factors strongly associated with an anxiety disorder in pregnancy (Bayrampour et al., 2018). A few studies have revealed that high resilience levels and high perceived social support may mitigate antenatal anxiety disorders (Ma et al., 2019; Peter et al., 2016).

Qatar is a high-income Arabic country located on the west coast of the Arabian Gulf and a member of the Gulf Cooperation Council. The Qatar National Health Strategy (2018–2022) focuses on preventative approaches among specific vulnerable cohorts such as pregnant women. In addition, the Primary Health Care Corporation (PHCC), the leading provider of preventive services aims for better population health, including mental health by 2023 (Ministry of Public Health, 2018).

**Figure 1:**
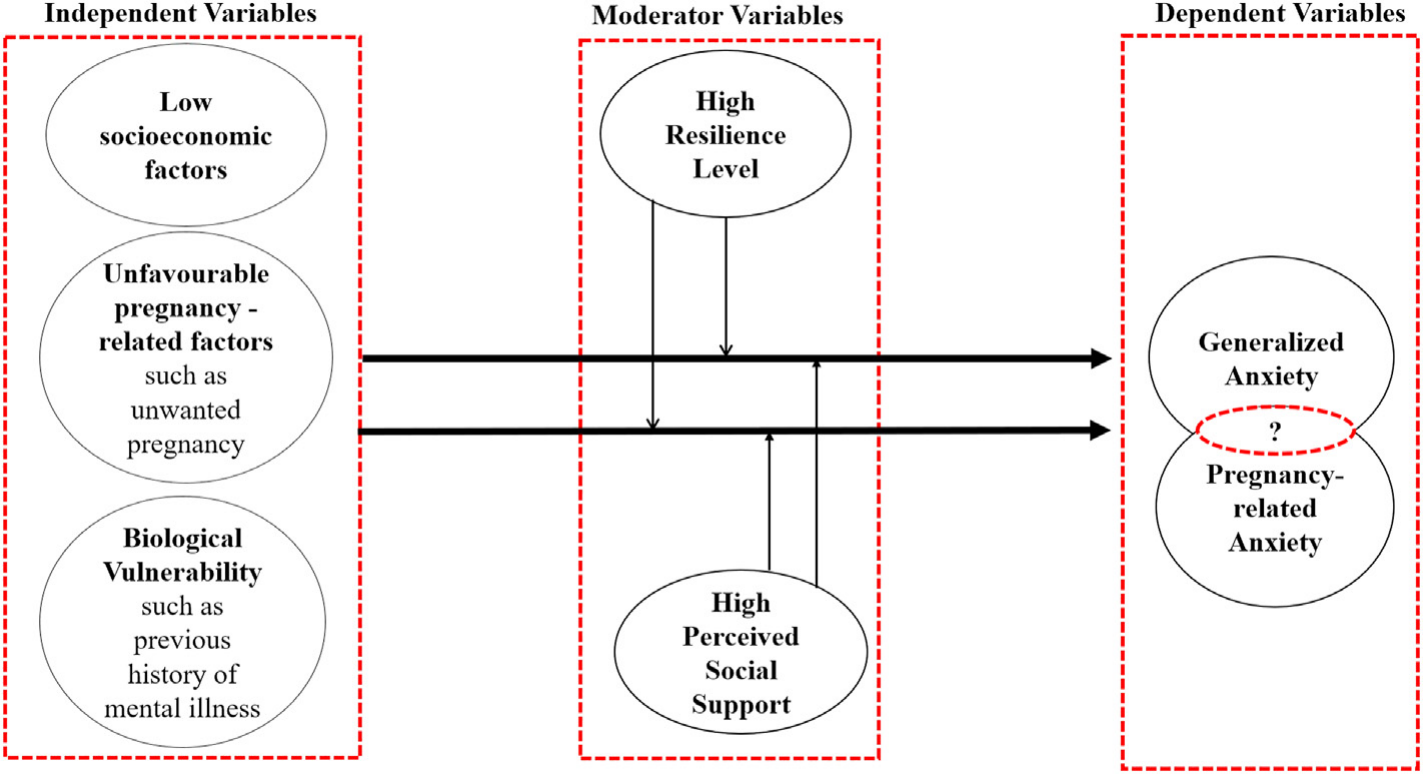
Conceptual framework of generalized and pregnancy-related anxiety among pregnant women.

The healthcare system did not implement antenatal anxiety screening program yet, which means that physicians are missing anxiety cases. Thus, policymakers do not know the prevalence of pregnancy-related concerns and generalized anxiety in Qatar. Accordingly, this study aims to determine the prevalence of generalized anxiety, pregnancy-related anxiety, and its determinants among Qatar's pregnant women. Consequently, we hypothesized that different antenatal anxiety types might be influenced by different determinants and predictors, building on the proposed conceptual framework in Figure 1.

## 2 Methods

### 2.1 Study design and setting

We conducted an analytical cross-sectional study among pregnant women attending their regular antenatal appointment of the Primary Health Care Corporation (PHCC) in Qatar. The data collection period was from September 2018 to February 2019. The PHCC provides preventive and free of charge services to the whole community in Qatar through twenty-three primary health care centers distributed across the country with antenatal participation rate as high as 60% (Primary Health Care Corporation, 2012).

### 2.2 Sampling technique

A cluster sampling technique was employed. Nine primary health care centers were selected randomly from the total (twenty-three at the time of the study), through the Automated Random Number Generator. Then the proportional allocation of sample size was computed based on the registered attendance at the health centers. We multiplied the number of registered pregnant women in each of the selected nine health centers by the total estimated sample size (*n* = 800), then we divided the outcome by the total number of pregnant women registered in the selected nine centers (*N* = 3766), as seen in Table 1. Later, we enrolled pregnant women who fulfilled the inclusion criteria in a non-random pattern until fulfilling the sample size (*n* = 800).

**Table 1:** Number of pregnant women selected from Primary Health Care Centers based on the proportionate allocation of the participants (*n* = 800).

Health Centers	Total(*N =* 3766)	Sample(*n* = 800)
1. Health Center 1	109	23
2. Health Center 2	450	96
3. Health Center 3	731	155
4. Health Center 4	664	141
5. Health Center 5	498	106
6. Health Center 6	262	56
7. Health Center 7	172	37
8. Health Center 8	440	93
9. Health Center 9	440	93

### 2.3 Sample size and participants enrolment

We determined the sample size through a given margin of error of 5% and a 95% Confidence Interval (*CI*) and an effect size of 50%. The outcome was multiplied by a design effect of two, estimating the sample size to be eight-hundred pregnant women (Charan and Biswas, 2013). The inclusion criteria involved all pregnant women of reproductive age (15–49 years old) who were willing to participate and communicate in Arabic or English. After granting a signed consent and assent, we interviewed the participants.

### 2.4 Materials

#### 2.4.1 Measurements tools related to dependent variables

The Pregnancy-Related Anxiety Revised Version Two (PRAQ-R2) is a second and shortened version of Pregnancy-Related Anxiety Questionnaire (PRAQ), which initially consisted of fifty-eight items (Van den Bergh, 1991). The ten-items Pregnancy-Related Anxiety Revised Version two (PRAQ-R2), originally in the English language, was used to measure the pregnancy-related anxiety level for all pregnant females irrespective of their parity. Each item asks about feelings at present and responses are measured on a 5-point Likert scale: absolutely not relevant, hardly ever relevant, sometimes relevant, reasonably relevant, and very relevant, coded as ‘0’, ‘1’, ‘2’, ‘3’, ‘4’, respectively. The total score ranged from 0 to 40. It consisted of three subscales: fear of giving birth, worries about a disabled child, and concern about one's appearance. This scale showed satisfactory internal consistency (*Cronbach's alpha* >0.8) (Huizink et al., 2016). The PRAQ-R2 was assessed by three studies with high methodological quality through the Cosmin checklist, concluding that substantial evidence shows that the PRAQ-R2 is an accurate indicator of pregnancy-specific anxiety (Sinesi et al., 2019).

The three-items Edinburgh Depression Scale (EDS-3A) is a self-administered subscale of the full ten-items EDS, and its total score ranges between 0 to 9 (Tuohy and McVey, 2008). The systematic review of anxiety scales revealed that the three items (3, 4 and 5) have consistent construct loading (>0.63, range 0.73–0.85) onto the anxiety subscale (Sinesi et al., 2019). The EDS-3A performed better than the anxiety subscale of the Hospital Anxiety and Depression Scale (HADS-A82) in detecting women with an anxiety disorder as determined by DSM diagnostic criteria (Zigmond and Snaith, 1983).

#### 2.4.2 Measurements tools related to independent variables

We developed an interview-based questionnaire to measure the independent variables. The questionnaire included questions on socio-demographic factors (marital status, age, educational level, employment, household income), medical history (gravity, number of children, gestational age, unplanned pregnancy, unwanted pregnancy, acute diseases that is any illness below three months, previous pregnancy-related complication, history of stillbirth) and history of mental illnesses.

#### 2.4.3 Measurements tools related to moderators variables

The Short Version of the Perceived Social Support Questionnaire (The F-SozU K-6), originally in the German language, was used to measure social support. The questionnaire consisted of six-items on a 5-point- Likert scale: Does not apply, Hardly Ever, sometimes apply, Reasonable apply, Exactly applicable, coded as ‘0’, ‘1’, ‘2’, ‘3’, ‘4’, respectively. The total score ranges from 0 to 24. It showed high internal consistency (*Cronbach's alpha* = 0.94). It is a self-administrated, reliable, and economically viable instrument to evaluate perceived practical, emotional, and social support systems. Practical, emotional, and social support integration refers to a sense of belonging (Kliem et al., 2015).

The Resilience Scale (RS-11) was used to measure the resilience level. Resilience is defined as the stable ability to modulate and control one's affective state and adequately adjust to burdens. RS-11 is the German short version of the Resilience Scale. It consists of eleven self-reported items on a 7-point- Likert scale (ranging from 1 ‘completely agree’ to 7 ‘completely disagree’), and its total score ranges from 7 to 77. It captures psychosocial stress-resistance. The authors reported high internal consistency (*Cronbach's alpha* = 0.91) (Schumacher et al., 2005).

#### 2.4.4 Procedure

After screening for eligibility criteria and signing the consent form, we interviewed the participants about their clinical history, including mental and medical history, in addition to pregnancy-related and socio-demographic characteristics. Subsequently, they disclosed their anxiety symptoms through EDS-3A and PRAQ-R2 tools and completed the resilience and perceived social support tools.

Two independent translators performed backward and forwarded translations of the tools from their original language to English and Arabic then backward from English to Arabic. The panel of experts evaluated the content validity of the measurement tools. Using Lawshe's method, the members rated each item for its importance and relevance by applying a 3-point rating scale: (1) not necessary, (2) useful but not essential, and (3) essential. The universal agreement between the three raters was 80% for the EDS and 90% for the PRAQ-R2. Finally, the tools were pre-tested to ensure their accuracy.

The EDS-3A tool showed acceptable internal consistency (*Cronbach's alpha* = 0.73), while the PRAQ-R2 scale showed higher internal consistency (*Cronbach's alpha* = 0.87). We computed the reliability of the Resilience Scale (RS-11) (Cronbach's alpha = 0.91) and the Perceived Social Support tool (FSoz-6) (Cronbach's alpha = 0.89).

#### 2.4.5 Ethical consideration

This study was approved by the Institutional Review Board of the Primary Health Care Corporation under protocol ID PHC/RC/18/04/002.

### 2.5 Analysis

Data analysis were carried out using the SPSS V.23 statistical package based on a pre-set significant level of .05. First, we conducted a descriptive analysis of the variables and presented the categorical outcome in frequency and percentages, while continuous variables were presented as mean and standard deviation. The dependent variables were dichotomized based on the 75th *percentile,* where it marked a cut-off of 13 for the pregnancy-related anxiety scale, a cut-off of 6 for subscale fear of giving birth, a cut-off of 5 for generalized anxiety scale, a cut-off of 4 for fear of having disabled child and concern about one's appearance. Second, we performed bivariate analyses using chi-square tests to compare the association between the dependent variables (pregnancy-related anxiety and generalized anxiety) and independent variables (sociodemographic, clinical characteristics, and others). Later, we performed a hierarchical regression analysis to examine the predictors' effects on generalized and pregnancy-related anxiety, the explained variance (R2), and the change in R2 (DR2). In the first step, we included the significant determinants in bivariate analyses, and second, we added the moderator terms (products) of resilience and perceived social support.

To examine the concordance and correlation of the two scales, we computed the Pearson correlation coefficient (r) for the PRAQ-R2 and EDS-3A tools. Additionally, we computed the scales' reliability through Cronbach's alpha (α).

Finally, using Stata, we did a confirmatory factor analysis on the PRAQ-R2 and EDS-3A tools to verify the factor load and test the goodness-of-fit.

## 3 Results

### 3.1 Study sample

Out of the 800 participants, the minority were Qatari (n = 145, 18.1%) and the rest non-Qatari. Regarding the occupation, less than half of the pregnant women were employed (n = 271, 33.8%). About one-thirds (n = 261, 32.6%) of the sample were primigravida. Approximately, half of the pregnant women were in their second trimester (n = 391, 48.9%). The participant's mean age was 28.8 ± 5 years. Most of the pregnant women (n = 792, 99 %) were married and only a few (n = 8, 1 %) were separated from their husbands.

### 3.2 Prevalence of general anxiety vs. pregnancy-related anxiety

**Figure 2:**
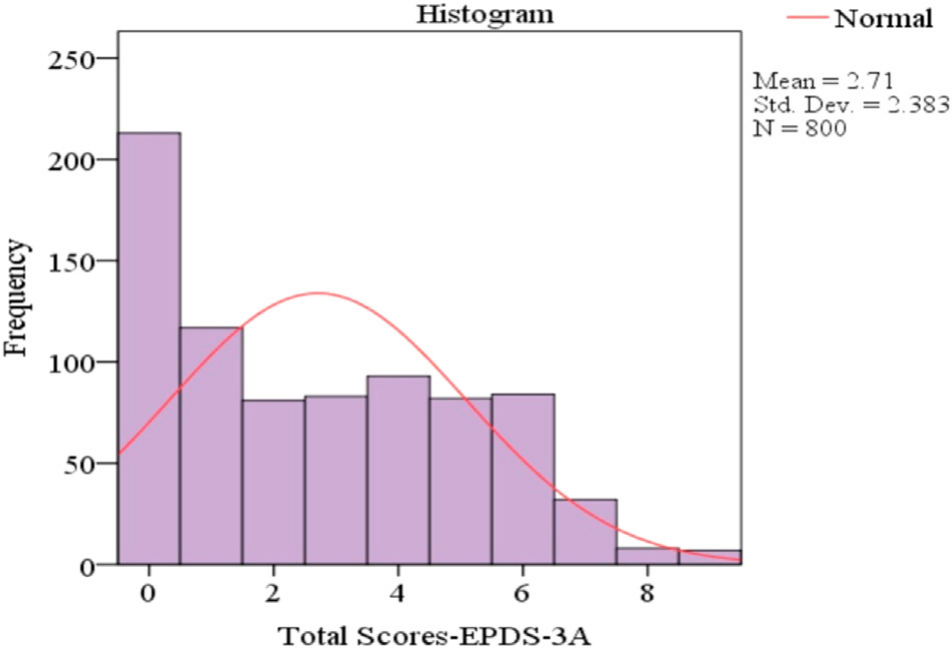
Distribution of the EDS-3A scores (n = 800).

Generalized and pregnancy-related anxiety were reported in (n = 83, 10.4%) of pregnant women. The proportion of pregnant women with positive screening results for generalized anxiety symptoms (EDS-3A≥5) was (n = 131,16.4%). The mean of EDS-3A score was 2.7, 95% *CI* [2.5–2.8]. The distribution of the scores reported by EDS-3A is presented in Figure 2.

**Figure 3:**
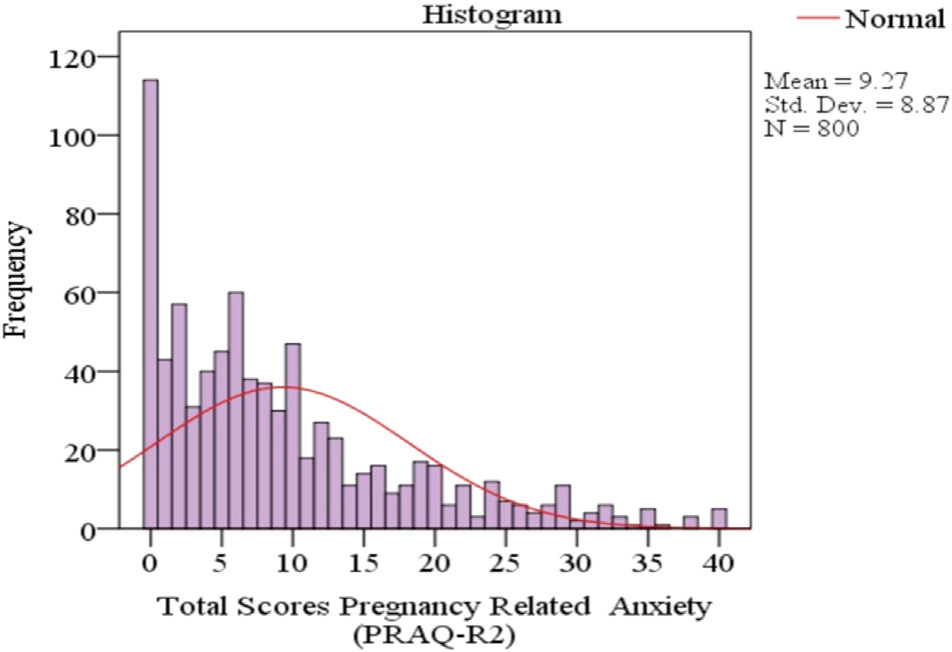
Distribution of the PRAQ-R2 scores (n = 800).

Out of the 800 pregnant women, a total of (n = 213, 26.6%) participants had a 13 or greater score on the PRAQ-R2, thus identified as having pregnancy-related anxiety. The mean score of PRAQ-R2 scores was 9.27, 95% *CI* [8.6–9.8]. Eighty-four participants reported fear of giving birth (n = 84,10.5%), sixty pregnant women described the fear of having a disabled child (n = 60,7.5%)—the least stated concern about one's appearance (n = 27,3.4%) of the participants. The distribution of the PRAQ-R2 scores is in Figure 3.

### 3.3 Correlation between EDS-3A and PRAQ-R2

The two scales showed a significant positive, but weak correlation demonstrated by the Pearson's Correlation Test (*r* = +0.32, *P* < 0.000).

### 3.4 Determinants of general anxiety vs. pregnancy-related anxiety

The socio-demographic factors, including age, nationality, occupation, monthly income, and educational level, failed to show any association with generalized and pregnancy-related anxiety. As seen in Table 2.

**Table 2:** Association of socio-demographic variables with generalized and pregnancy-related anxiety (*n* = 800).

Socio-demographic Variables	Generalized Anxiety EDS-3A (Score>5)				Pregnancy-related Anxiety PRAQ-R2 (Score ≥13)			
	*Yes n (%)*	*No n (%)*	*χ*^2^	*OR [95%CI]*	*Yes n (%)*	*No n (%)*	χ^2^	*OR [95%CI]*
**Age group (Full years)**								
≤19	7 (3)	17 (3)	2	1.18 [ 0.79–1.75]	5 (2)	19(3)	1	0.97 [ 0.64–1.47]
20–34	170(80)	491(84)			176 (83)	485(83)		
35–46	36(17)	79(13)			32 (15)	83(14)		
**Nationality**								
Qatari	39 (18)	106(18)	0	1.07 [ .68–1.51]	46 (22)	99(17)	2	1.35 [0.91–2]
Non-Qatari	174(82)	481(82)			167 (78)	488(83)		
**Occupation**								
Housewife	121 (57)	363 (62)	7	1.05 [ 0.72–1.38]	134 (63)	350(60)	3	.71 [0.52–1]
Employed	85 (40)	186 (32)			72 (34)	199(34)		
Students	7 (3)	38 (6)			7 (3)	38 (6)		
**Monthly income (QR)**								
<20000	40 (19)	130(22)	2	1.07 [0.85–1.35]	44 (21)	126(22)	1	1.11 [ 0.87–1.39]
10,000–20,000	79 (37)	194(33)			77(36)	196(33)		
Up to 10,000	94 (44)	263(45)			92 (43)	265(45)		

*Note*: EDS-3A = Edinburgh Depression Scale-three items Anxiety Scale; PRAQ-R2 = Pregnancy-Related Anxiety Revised Version two; *CI* = Confidence Interval; QR = Qatari Ryal; ∗p < 0.05; ∗∗p ≤ 0.0001; χ^2^*=* Chi-square; *OR = Odd Ratio*.

Some pregnancy-related characteristics, such as unwanted pregnancy and previous stillbirth, were statistically associated with generalized and pregnancy-related anxiety. However, unplanned pregnancy and current illness were only associated with generalized anxiety. The previous history of mental illness was shown to be statistically associated with generalized and pregnancy-related anxiety, increasing the odds of having generalized anxiety 20-fold and increasing the probability of having pregnancy-related anxiety by 15 times. However, low perceived social support and resilience levels were inversely associated with generalized and pregnancy-related anxiety, as seen in Table 3.

**Table 3:** Association of pregnancy-related variables with generalized and pregnancy-related anxiety(n = 800).

Pregnancy -related variables	Generalized Anxiety EDS-3A (score>5)				Pregnancy-related anxiety PRAQ-R2(score>13)			
	Yes n (%)	No n (%)	χ^2^	*OR [95%CI]*	Yes n (%)	No n (%)	*χ*^2^	*OR [95%CI]*
**Trimesters**								
1^st^	60 (28)	137(23)	3	0.93 [0.75–1.16]	58(27)	139(24)	2	0.99 [0.79–1.24]
2^nd^	95 (45)	296(51)			95(45)	296(50)		
3^rd^	58 (27)	154(26)			60(28)	152 (26)		
**Gravity**								
Primigravida	65 (30)	196 (33)	1	0.87 [0.62–1.22]	71(33)	190 (32)	0	1 [0.74–1.45]
Multigravida	148 (70)	391 (67)			142(67)	397 (68)		
**Previous stillbirth**								
Yes	10 (5)	5(1)	13	5.73[1.93–16.97] ∗∗	8 (4)	7(1)	6	3.23[1.15–9.02] ∗
No	203 (95)	582(99)			205 (96)	580(99)		
**Unplanned Pregnancy**								
Yes	100(47)	383(65)	10	1.66[1.21–2.28] ∗∗	81 (38)	223 (38)	0	1[0.72–1.38]
No	113(53)	204(35)			132 (62)	364 (62)		
**Unwanted Pregnancy**								
Yes	23 (89)	12(2)	29	6[2.83–11.88] ∗∗	21(90)	14(2)	21	4.47[2.23–8.97] ∗∗
No	190(11)	575(98)			192(10)	573(98)		
**Current illness**								
Yes	133(62)	283(48)	13	2 [1.29–2.46] ∗∗	119(56)	297(51)	2	1.23 [0.91–1.69]
No	80 (38)	304(52)			94 (44)	290(49)		
**Previous history of mental illness**								
Yes	200(94)	5 (1)	20	8[2.66–21.48] ∗∗	12(6)	6(1)	15	6[2.14–15.61] ∗∗
No	13 (6)	582(99)			201(94)	581(99)		
**Perceived Social Support**								
Low	49(23)	46(8)	34	0.28[0.18–0.44] ∗∗	54 (25)	41(7)	50	0.22 [0.14–0.34] ∗∗
High	164(77)	541 (92)			159 (75)	546(93)		
**Resilience level**								
Low	160 (75)	334 (57)	22	0.43[0.30–0.62] ∗∗	156(73)	338(58)	16	0.49[0.35–0.70] ∗∗
High	53 (25)	253 (43)			57(27)	249(42)		

*Note*: EDS-3A = Edinburgh Depression Scale-three items Anxiety Scale; PRAQ-R2 = Pregnancy-Related Anxiety Revised Version two; *CI* = Confidence Interval; ∗p < 0.05; ∗∗p ≤ 0 .0001; χ^2^ = Chi-square; OR = Odd Ratio.

### 3.5 Predictors of general anxiety vs. pregnancy-related anxiety

The probability of having generalized anxiety increases in the presence of a history of stillbirth, unwanted pregnancy, current illness, and mental illness. The moderating effect of perceived social support and resilience level was statistically significant for generalized anxiety. The goodness-of-fit tests are all below the significance level of 0.05, which indicates that there is enough evidence to conclude that the models fit the data. We present the logistic regression results of generalized anxiety predictors in Table 4.

**Table 4:** Predictors of generalized anxiety among pregnant women through the application of Binary Logistic Regression (*n* = 800).

	Generalized Anxiety EDS-3A(Score> 5)					
	Step1			Step 2		
	*Beta coefficient*	*Exp(B)*	*95% CI of Exp (B)*	*Beta Coefficient*	*Exp(B)*	*95%CIof Exp(B)*
Previous stillbirth	1.69	5.46	[1.76–16.87] ∗	1.48	4.39	[1.31–13.85] ∗
Unplanned pregnancy	0.35	1.42	[1.01–2] ∗	0.36	1.43	[1.02–2.02] ∗
Unwanted pregnancy	1.35	3.86	[1.81–8.27] ∗∗	1.13	3.11	[1.41–6.85] ∗
Current illness	0.52	1.67	[1.21–2.34] ∗	.48	1.62	[1.15–2.26] ∗
Previous history of mental illness	1.71	5.47	[1.84–16.25] ∗	1.62	5.09	[1.68–15.36] ∗
Moderator Variables (RS x PS)	…	…	…	0.136	1.14	[1.01–1.28] ∗
Constant	-1.59	0.2∗∗	…	-1.61	0.19∗∗	

Step 1: Cox & Snell R square = 0.07; Nagelkerke R square = 0.10.

Step 2: Cox & Snell R square = 0.08; Nagelkerke R square = 0.12.

*Note*. Exp (B) = Exponentiation of the B coefficient; RS = Resilience; PS = Perceived Social Support; ∗P value ≤ .05; ∗∗P value ≤.0001; EDS-3A = Three items of Edinburgh Depression Scale; CI = Confidence Interval.

Similar to generalized anxiety, the presence of a history of stillbirth, unwanted pregnancy, current illness, and mental illness predicts pregnancy-related anxiety. However, unplanned pregnancy and current illness failed to show a significant association with pregnancy-related anxiety. The moderating effect of perceived social support and resilience level on pregnancy-related anxiety was statistically significant. The goodness-of-fit tests were all below the significance level of 0.05, which indicates that there is enough evidence to conclude that the models fit the data, as seen in Table 5.

**Table 5:** Predictors of pregnancy-related anxiety among pregnant women through the application of Binary Logistic Regression (*n* = 800).

Predictors	Pregnancy-related anxiety PRAQ-R2 (Score ≥13)					
	*Step 1*			*Step 2*		
	*Beta coefficient*	*Exp(B)*	*95% CI of Exp (B)*	*Beta coefficient*	*Exp(B)*	*95% CI of Exp (B)*
Previous stillbirth	1.03	2.81	[.97–8.14]	0.67	1.96	[0.63–6.11]
Unwanted pregnancy	1.35	3.88	[1.92–7.94] ∗	0.99	2.71	[1.25–5.86] ∗
Current medical illness	0.13	1.14	[0.82–1.51]	0.06	1.07	[0.77–1.42]
Previous history of mental illness	1.47	4.37	[1.56–12.21] ∗	1.33	3.79	[1.31–11.01] ∗
Moderator Variables (RS x PS)	…	…	…	0.26	1.29	[1.15–1.46] ∗∗
Constant	-1.22	0.29∗∗	…	-1.26	0.28∗∗	…

Step1: Cox & Snell R square = 0.04; Nagelkerke R square = 0.05.

Step 2: Cox & Snell R square = 0.06; Nagelkerke R square = 0.09.

*Note*. Exp (B) = Exponentiation of the B coefficient; RS = Resilience; PS = Perceived Social Support; ∗P value ≤ .05; ∗∗P value ≤.0001; CI = Confidence Interval.

### 3.6 Confirmatory factor analyses

Table 6 shows the outcome of confirmatory factor analysis, observed variables (items 1,2,4,5,6,7,8,9, and 10) of the pregnancy-related anxiety scale, and the latent variable (anxiety). The p-values for all the factor loadings are statistically significant, below the typical cut-off point of 0.05. Item 8 has the most vital loading of the items with a standardized factor loading of 0.9; meaning that a one standard deviation increase in anxiety leads to a 0.9 standard deviation increase in the response to the question. The weakest measure of the parameter is item 1.

**Table 6:** Confirmatory factor analysis with factor loadings.

Variables	Standardized Factor loading	OIM Coef.	St. Err	P>|z|	P value	[95%Confidence Interval]
Fear of giving birth	Item 1 ‘I am anxious about the delivery’	0.42	0.30	13.93	0.000	[0.36–0.48]
	Item 2 ‘I am worried about the pain of contractions and the pain during delivery’	0.56	0.26	21.58	0.000	[0.51–0.61]
	Item 6 ‘I am worried about not being able to control myself during labour and fear that I will scream’	0.57	0.02	22.32	0.000	[0.52–0.62]
Concerned about appearance	Item 5 ‘I am concerned about my unattractive appearance’	0.43	0.03	14.22	0.000	[0.37–0.49]
	Item 7 ‘I am worried about my enormous weight gain’	0.54	0.02	20.18	0.000	[0.48–0.59]
Concerned about baby health	Item 4 ‘I sometimes think that our child will be in poor health or will be prone to illnesses’	0.80	0.01	54.9	0.000	[0.51–0.83]
	Item 8 ‘I am afraid the baby will be mentally handicapped or will suffer from brain damage’	0.90	0.00	101.03	0.000	[0.89–0.92]
	Item 9 ‘I am afraid our baby will be stillborn, or will die during or immediately after delivery’	0.88	0.01	85.18	0.000	[0.85–0.90]
	Item 10 ‘I am afraid that our baby will suffer from a physical defect or worry that something will be physically wrong with the baby’	0.57	0.02	22.55	0.000	[0.52–0.62]

Log likelihood = -10546.44; number of observations = 800.

Likelihood test of model vs. Saturated: chi2 (27) = 837.29, Prob > Chi2 = .000.

Root mean squared error of approximation (RMSEA) = .14[.21-.18], Probability REMSA≤ .05.

Comparative fit index (CFI) = .78.

SRMR = Standardized root mean squared residual = .05.

Overall R2 = .92.

AIC = Akaike's information criterion = 21146.89.

BIC = Bayesian information criterion = 212273.

## 4 Discussion

This is the first study to examine anxiety disorders among pregnant women in Qatar. The results showed that the prevalence of pregnancy-related anxiety in Qatar (n = 213, 26.6%) is higher than the prevalence of generalized anxiety(n = 131,16.4%).

The prevalence of pregnancy-related anxiety in our sample is similar to the reported prevalence in Saudi Arabia (n = 195,23.6%) (Alqahtani et al., 2018). Our results suggest that the prevalence of pregnancy-related anxiety reported in Qatar is higher than in a neighbouring Arab country, Kuwait (n = 279,15%) (Pampaka et al., 2018) as well as being higher than Tanzania (n = 53, 25%) (Wall et al., 2018). The comparison is relevant as both studies used the same measurement tool and representative sample. However, the prevalence of pregnancy-related anxiety yielded by our study is much lower than that reported in India (n = 136, 55.7%). The discrepancy in the percentages reported may be due to the utilization of a less specific screening tool ‘RRT scale’ in India that may overestimate the prevalence of anxiety (Nath et al., 2019).

Regarding the subscales, fear of giving birth is more prevalent than the stress of having a disabled child or concerns about one's appearance. Fear of giving birth in our participants (n = 84, 10.5%) was higher than for pregnant women in Iran (n = 32, 6.1%) (Mortazavi and Agah, 2018). In contrast, our percentage is lower than the prevalence disclosed in Ireland (n = 324, 36.7%) (O Connell et al., 2019).

Our study revealed generalized anxiety to affect (n = 131, 16.4%) when assessed through EDS-3A. This is similar to the data reported in Canada (n = 49, 15.8%) (Fairbrother et al., 2016) and South London (n = 93, 17%) (Nath et al., 2018). However, the prevalence of generalized anxiety assessed through EDS-3A was revealed to be higher than the prevalence reported through conducting diagnostic interviews DSMIV as (n = 265, 9.5%) in Australia (Buist et al., 2011) and (n = 8, 2%) in South Africa (Van Heyningen et al., 2017). The inconsistency in the proportion of women who report generalized anxiety in previous studies could be due to utilizing a screening tool that tends to produce high false positives rates (Matthey et al., 2013).

Pregnancy-related anxiety and generalized anxiety shared some determinants. Specifically, parity failed to show any significant association with the two types of antenatal anxiety. This finding contrasts with the results of a longitudinal study that revealed a significant association between parity and antenatal anxiety (Blackmore et al., 2016). Furthermore, the previous history of mental illness and unwanted pregnancy was shown to be statistically associated with both types of anxiety, in line with the systematic review (Bayrampour et al., 2018) conducted between 2003 and 2015 involving ninety-seven scientific papers. Additionally, resilience and perceived social support were shown to be moderating variables, inversely associated with antenatal anxiety (Ma et al., 2019; Peter et al., 2016).

Our result revealed different predictors for the two types of anxiety; acute illness, and unplanned pregnancy succeeded in predicting generalized anxiety but failed to predict pregnancy-related anxiety. Therefore, different determinants and predictors were shown to influence antenatal anxiety types (generalized and pregnancy-related anxiety), which allow us to reject the null hypothesis.

The Pearson Correlation revealed a significant but weak positive correlation between the two scales (r = +0.32, P < 0.000). The shared variance is low; this indicates that generalized anxiety and pregnancy-related anxiety measure similar but distinct constructs. Approximately one in ten participants (n = 83, 10.4%) were found to suffer from both types of anxiety. This finding is in line with a longitudinal study that showed a moderate association between pregnancy-related anxiety and generalized anxiety (Blackmore et al., 2016).

This study adds essential value to the current literature since it is the first to assess pregnancy-related anxiety and generalized anxiety prevalence and predictors among Qatar's pregnant women. We utilized the probability sampling technique, and the sample size is large enough to allow generalization of the results, therefore, preserving external validity. Moreover, we conducted a Logistic Regression to deal with confounding variables. We followed specific strategies to prevent measurement bias; we did content and face validity for the measurement tools. Then, we performed the internal consistency of the measurement tools.

### 4.1 Limitations

This study has some limitations. First, it is essential to bear in mind that the EDS-3A and the PRAQ-R2 scales are self-report tools with no established cut-off point offering overestimated prevalence. The ideal situation would be to have a clinical interview. Second, the cross-sectional design lacks the temporal relationship that compromises one item of causality.

## 5 Conclusions

In conclusion, the current study showed that pregnancy-related anxiety is more prevalent than generalized anxiety in pregnancy. Interestingly, different predictors influenced generalized and pregnancy-related anxiety. Healthcare providers must screen for pregnancy-related anxiety and ask pregnant women about the history of mental illness. Policymakers must provide tailored interventions to increase resilience and perceived social support to prevent pregnancy-related anxiety and generalized anxiety.

## Declarations

### Author contribution statement

S. Naja: Conceived and designed the experiments; Performed the experiments; Analyzed and interpreted the data; Contributed reagents, materials, analysis tools or data; Wrote the paper.

N. Al Kubaisi: Conceived and designed the experiments; Performed the experiments; Wrote the paper.

R. Singh: Analyzed and interpreted the data; Contributed reagents, materials, analysis tools or data; Wrote the paper.

I. Bougmiza: Conceived and designed the experiments; Analyzed and interpreted the data; Wrote the paper.

### Funding statement

Open Access funding provided by the Qatar National Library.

### Competing interest statement

The authors declare no conflict of interest.

### Additional information

No additional information is available for this paper.
